# Dynamics of Microbial Inactivation and Acrylamide Production in High-Temperature Heat Treatments

**DOI:** 10.3390/foods10112535

**Published:** 2021-10-21

**Authors:** Jose Lucas Peñalver-Soto, Alberto Garre, Arantxa Aznar, Pablo S. Fernández, Jose A. Egea

**Affiliations:** 1 Departamento de Ingeniería Agronómica, Instituto de Biotecnología Vegetal, Universidad Politécnica de Cartagena (ETSIA), Paseo Alfonso XIII, 48, 30203 Cartagena, Spain; joselucasps@gmail.com (J.L.P.-S.); arantxa.aznar@upct.es (A.A.); pablo.fernandez@upct.es (P.S.F.); 2 Centro de Edafología y Biología Aplicada del Segura (CEBAS-CSIC), Campus Universitario de Espinardo, 30100 Murcia, Spain; 3 Food Microbiology, Wageningen University & Research, P.O. Box 17, 6700 AA Wageningen, The Netherlands; alberto.garreperez@wur.nl

**Keywords:** food safety, acrylamide formation, thermal resistance, dynamic models, simulation

## Abstract

In food processes, optimizing processing parameters is crucial to ensure food safety, maximize food quality, and minimize the formation of potentially toxigenic compounds. This research focuses on the simultaneous impacts that severe heat treatments applied to food may have on the formation of harmful chemicals and on microbiological safety. The case studies analysed consider the appearance/synthesis of acrylamide after a sterilization heat treatment for two different foods: pureed potato and prune juice, using *Geobacillus stearothermophilus* as an indicator. It presents two contradictory situations: on the one hand, the application of a high-temperature treatment to a low acid food with *G. stearothermophilus* spores causes their inactivation, reaching food safety and stability from a microbiological point of view. On the other hand, high temperatures favour the appearance of acrylamide. In this way, the two objectives (microbiological safety and acrylamide production) are opposed. In this work, we analyse the effects of high-temperature thermal treatments (isothermal conditions between 120 and 135 °C) in food from two perspectives: microbiological safety/stability and acrylamide production. After analysing both objectives simultaneously, it is concluded that, contrary to what is expected, heat treatments at higher temperatures result in lower acrylamide production for the same level of microbial inactivation. This is due to the different dynamics and sensitivities of the processes at high temperatures. These results, as well as the presented methodology, can be a basis of analysis for decision makers to design heat treatments that ensure food safety while minimizing the amount of acrylamide (or other harmful substances) produced.

## 1. Introduction

Different conflicting objectives often arise in many food processes (e.g., quality vs. economical cost). Finding the optimal solutions which balance among all the existing objectives is not an easy task due to the complexity of the mathematical models describing such processes [1,2,3,4]. This optimization step is crucial to produce an efficient decision-making process [5]. Recent research has been devoted to optimizing food processes where two or more conflicting objectives appear. The most common are usually related to product quality and process economy [6,7,8], different quality parameters [9,10,11], economic and environmental parameters [12,13], or, as in the present study, product quality and safety [14]. 

One of the most important methods of food preservation in the food industry is thermal processing. Historically, the focus was on optimizing heat treatments to improve the processes related to microbial destruction, nutrients retention, cooking values and loss of quality [15]. The main function of heat treatments is to inactivate microorganisms and enzymes to achieve safe, long shelf-life food. The associated disadvantages are related to food quality (e.g., nutrients or texture preservation) due to the effect of high temperatures. Therefore, the design of thermal processes in the food safety sector must face different objectives, such as food quality or energy consumption vs. microbial inactivation. The use of high temperatures implies food degradation but also the formation of substances that can be harmful to humans [16]. One example is acrylamide, a chemical that is produced during high-temperature processes in foods that contain reducing sugars (such as fructose and glucose) and asparagine [17]. Acrylamide was found to be carcinogenic in rodents, and the International Agency for Research on Cancer has classified it as a probable carcinogen [18,19]. This has motivated food authorities to propose methodologies to minimize acrylamide content in commercial and homemade foods. The European Food Safety Authority (EFSA) has set recommendation levels for some foods [20]. The influence of high temperature on the formation of acrylamide has been previously demonstrated [21]. The higher the processing temperature, the more acrylamide is formed, thus heat treatments need to be optimized to decrease the amount of produced acrylamide. The formation of acrylamide in a process that involves the heating of food is explained by the Maillard reaction [22]. Since its formulation, this reaction has been studied from different points of view. Traditionally, the focus was put on components that affected colour, flavour, and taste, whereas more recently the focus has moved to the analysis of the formation of mutagens and carcinogens. Acrylamide is one of these chemicals in the spotlight due to its potential formation in highly consumed foods such as potato chips [23] or Asian noodles [24]. French fries, coffee, and bread have also presented high levels of acrylamide [21]. In fact, a wide range of different food products containing fructose and asparagine can contain acrylamide. 

In the case of baby food, the recommendations for acrylamide levels are more restrictive than in other types of food, and a maximum allowed amount of 30 μg/kg is set [20]. Different studies [25,26] have shown that various foods exceed these limits. Specifically, potato-based products are highly susceptible to containing high levels of acrylamide. On the other hand, although the EFSA has not yet established recommendations for foods based on vegetables or fruits [27], it has been shown that products such as prune juice can contain high concentrations of acrylamide, reaching much higher values even than in potato-based foods. [28]. Therefore, this study focuses on foods such as potato puree and prune juice, which can be catalogued as baby food and may not meet EFSA’s recommendation for those products.

From a quality vs. microbiological point of view, heat stability of heat-labile quality factors presents a higher z-value than those typical of bacteria. Then, high-temperature short-time (HTST) processes are less deleterious to food quality while ensuring microbial food safety and stability [29], although the impact of acrylamide formation has not been considered. In this regard, the two proposed objectives (i.e., microbial inactivation and acrylamide formation) are opposed and the problem must be analysed.

The microorganism considered here is *Geobacillus stearothermophilus*, a Gram-positive, thermophilic, and spore-forming bacterium with an optimal growth temperature around 55 °C. The spores are very heat-resistant and usually survive canning and sterilization operations. Furthermore, it has been detected in different foods such as canned vegetables, ready-to-eat meals containing meat, fruit preparations, or dehydrated ingredients [30]. Other relevant pathogenic spore formers of interest in the food industry, such as *Clostridium botulinum* or *Bacillus cereus*, have not been considered here, as their inactivation is generally not a problem within the range of temperatures considered in this study which give rise to significant amounts of formed acrylamide.

As *Geobacillus stearothermophilus* spores are used to validate heat sterilization processes [31,32,33,34], this study evaluates the inactivation of this microorganism in a heat treatment within the typical temperatures applied to the studied products (120–135 °C).

In this work, we have simulated and analysed the dynamics of the two objectives in a thermal inactivation operation. The aim is to use mathematical models to determine the conditions where the amount of formed acrylamide is minimum while ensuring microbial inactivation. To the authors’ knowledge, no previous work was published facing the inactivation of microorganisms and acrylamide production.

## 2. Materials and Methods

### 2.1. Case Study

This study analyses the dynamics of two processes associated with the application of severe heat treatments in some foods: microbial inactivation and acrylamide production. In principle, higher temperatures ensure a safer operation from the microbiological point of view, but can also induce a higher acrylamide production. We have applied this analysis to two practical cases of the food industry (potato puree and prune juice) where different time/temperature treatments are simulated to assess the inactivation of a thermo-resistant microorganism and the acrylamide formation. The microorganism is first characterized (see Section 2.2) and then heat treatments scenarios are simulated for each of the considered foods to assess the dynamics of microbial inactivation and acrylamide formation and their balance.

### 2.2. Microbial Inactivation Model and Parameter Estimation

To characterize the behaviour of the microorganism, the Bigelow model [35] was chosen. This choice is motivated by the use of this model to characterize the microbial inactivation of *Geobacillus stearothermophilus* within the literature (e.g., [36,37,38]). In any case, the use of other models would not invalidate the methodology presented here, and the whole procedure would be similar. It considers a log-linear relationship between the fraction of survivors (S) and treatment time, (t), as shown in Equation (1).

(1) $$log_{10}S=\frac{-t}{D\left(T\right)}$$

(2) $$log_{10}D\left(T\right)=log_{10}D\left(T_{ref}\right)-\frac{T-T_{ref}}{z}$$

The influence of treatment temperature $\left(T\right)$ in the microorganism is reflected as the D-value, which is log dependent on the temperature, as shown in Equation (2). The D-value represents the time required to reduce the microbial population by 90% at a constant temperature, and the z-value quantifies the sensitivity of the D-value to temperature changes. The reference temperature, $T_{ref}$, is a parameter without biological meaning but can improve parameter identifiability [39,40]. 

To estimate the model parameters, several D-values (n = 113) for thermal inactivation of *Geobacillus stearothermophilus* were collected from the literature (Web of Science database) as described in [41]. A temperature range between 97.5 °C and 137.5 °C was considered, and only food matrixes (especially vegetable-based) were included [38,42,43,44,45,46,47,48,49,50]. Non-linear regression was applied to obtain mean $log_{10}D\left(T_{ref}\right)$-values and z-values with their respective standard errors.

The estimated model parameters are $log_{10}D\left(T_{ref}\right)=-0.0468\pm 0.03789$, z=8.66±0.283 (°C). The experimental data, as well as the fitted model, are presented in Figure S1, that shows a good model fitting, which confirms the suitability of the Bigelow model to characterize the microorganism in the conditions considered. The reference temperature was set to a value near the middle of the temperature range ($T_{ref}=125$ °C) as recommended by [40]. Monte Carlo simulations were used to calculate the probability that a heat treatment (time, temperature) produces at least 6 logarithmic reductions in the microbial load (symbolized as $P\left(log_{10}S\geq 6\right)$ within the text), which is considered to be sufficient for many inactivation processes. In any case, changing this value would not change the proposed methodological approach. For this, we select 1000 pairs of values of the model parameters $\left(log_{10}D\left(T_{ref}\right),z\right)$ obtained by simulation from two independent normal distributions, where $log_{10}D\left(T_{ref}\right)\;\sim \;N\left(\mu =-0.0468,\sigma =0.03789\right)$ and $z\;\sim \;N\left(\mu =8.66,\sigma =0.283\right)$. Next, the expected log reduction was calculated with Equations (1) and (2) from the heat treatment conditions (time, temperature) for each of the 1000 pairs of parameter values. Finally, the probability of achieving the target inactivation was calculated by dividing the number of cases that comply with $log_{10}S\geq 6$ by 1000. This procedure was used to address the first objective: maximize $P\left(log_{10}S\geq 6\right)$.

### 2.3. Acrylamide Production Objective

To quantify the acrylamide formation, the multi-response kinetics in a glucose-asparagine reaction at high temperatures (120–200 °C) proposed by [51] were used. The model is based on the reaction network shown in Figure S2. 

Glucose and asparagine react to form a Schiff base. Fructose is formed by glucose isomerization, and it also reacts with asparagine to form the Schiff base. At the same time, the Schiff base is degraded into melanoidins and acrylamide, whereas acrylamide is degraded into unknown species (named *Product X*). Study [51] calculated the equilibrium constants for the temperature range (120–200 °C), which showed a logarithmic relationship with temperature. Using this observed relationship, a temperature-dependent function was fitted for each constant ($K_{i}\left(T\right)\forall i=1,2\ldots ,6$). Although the range of temperatures used by Knol et al. does not coincide with the range considered for t$log_{10}D\left(T\right)$ vs. *T* values in this study (e.g., from 97.5 to 137.5), we consider that the same logarithmic relationship applies in our case. The estimated kinetic constant values were used in the system of ordinary differential equations that quantifies the amount of acrylamide formed for a specific time and temperature, shown in Equation (3).

(3) $$\left\{\begin{array}{c} \frac{d\left[Glucose\right]}{dt}=-K_{1}\left(T\right)\cdot \left[Glucose\right]\cdot \left[Asparagine\right]-K_{2}\left(T\right)\cdot \left[Glucose\right]\\ \frac{d\left[Fructose\right]}{dt}=-K_{3}\left(T\right)\cdot \left[Fructose\right]\cdot \left[Asparagine\right]+K_{2}\left(T\right)\cdot \left[Glucose\right]\\ \frac{d\left[Asparagine\right]}{dt}=-K_{1}\left(T\right)\cdot \left[Glucose\right]\cdot \left[Asparagine\right]-K_{3}\left(T\right)\cdot \left[Fructose\right]\cdot \left[Asparagine\right]\\ \frac{d\left[Schiffbase\right]}{dt}=K_{1}\left(T\right)\cdot \left[Glucose\right]\cdot \left[Asparagine\right]+K_{3}\left(T\right)\cdot \left[Fructose\right]\cdot \left[Asparagine\right]\\ -K_{4}\left(T\right)\cdot \left[Schiffbase\right]-K_{5}\left(T\right)\cdot \left[Schiffbase\right]\\ \frac{d\left[Acrylamide\right]}{dt}=K_{4}\left(T\right)\cdot \left[Schiffbase\right]-K_{6}\left(T\right)\cdot \left[Acrylamide\right] \end{array}\right.$$

One of the solutions to the system of differential equations is the acrylamide concentration formed for a certain heat treatment (time, temperature), which is the second objective in our formulation. In this second objective, apart from the heat treatment conditions, that is, the time and temperature variables, it is necessary to set the initial amounts of glucose, fructose, and asparagine. Two foods were selected: potato puree (typical pH of 5.1–6.0) and prune juice (typical pH of 4.0–5.0). For each of the two selected foods, initial concentrations are shown in Table 1. Details on these calculations are provided in Supplementary Material. From the existing potato varieties, the *red potato* was chosen, as it produces the highest amount of acrylamide [52].

## 3. Results and Discussion

This section analyses the results of the simulation of isothermal heat treatments with temperatures between 120 and 135 °C for two food matrices, pureed potatoes and prune juice. On the one hand, to quantify inactivation, a Monte Carlo simulation approach is used, as explained in Section 2.2, which provides the probability that the heat treatment (time, temperature) produces at least six logarithmic reductions ($P\left(log_{10}S\geq 6\right)$). Therefore, all results for inactivation in both foods are related to that probability. 

On the other hand, acrylamide production is reported in the units of measurement $\left(\frac{\mu g}{kg}\right)$ recommended by EFSA to make comparisons [20].

### 3.1. Single-Objective Analysis

**Figure 1:**
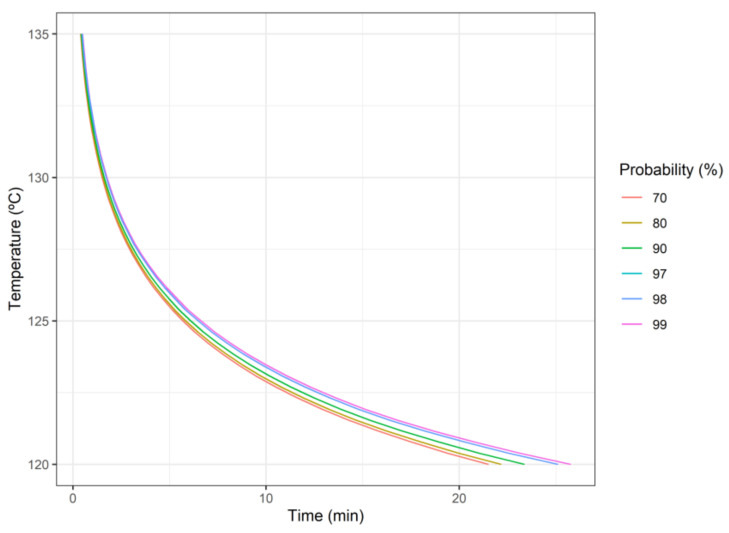
Probability of six or more logarithmic reductions $P\left(log_{10}S\geq 6\right)$ for *Geobacillus stearothermophilus* as a function of heat treatment (time, temperature).

Regarding the first objective (i.e., microbial inactivation), $P\left(log_{10}S\geq 6\right)$ as a function of temperature and time of heat treatment is presented in Figure 1. As expected, the areas with the highest probability of having more than six log-reductions in the microbial count are those related to higher temperatures and longer treatment times. It is notable that, in the ranges of time and temperature considered (Figure 1), $P\left(log_{10}S\geq 6\right)$ is very sensitive to small changes in temperature or in processing time. This sensitivity is higher as the temperature increases.

**Figure 2:**
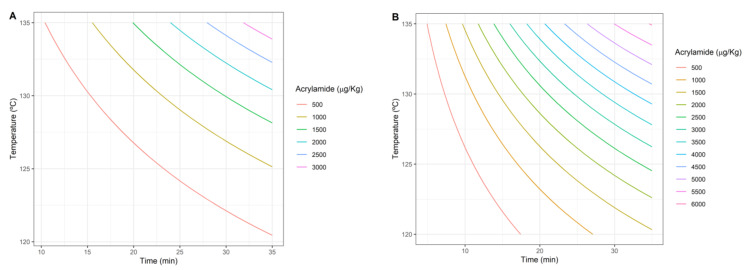
Amount of acrylamide formed as a function of heat treatment (time, temperature) for the potato puree (**A**) and prune juice (**B**).

On the other hand, the impact of the heat treatment on acrylamide formation is represented in Figure 2A for pureed potato and Figure 2B for prune juice. The area showing the highest acrylamide formation is defined by the highest treatment temperature. As expected, higher temperature and/or longer duration of the heat treatment had a positive correlation with acrylamide formation, although significant differences were found between the foods tested. The main difference was the amount of acrylamide that could be produced, which was higher for prune juice at all the time/temperature combinations. For example, for the most severe treatments (upper right corner of Figure 2A,B) the concentration was around two times higher in the case of prune juice. Comparing the isolines of both objectives, larger changes in temperature or processing time are needed to produce significant changes in acrylamide production (Figure 2) than in $P\left(log_{10}S\geq 6\right)$ (Figure 1). In other words, acrylamide formation is less sensitive to temperature and time than inactivation.

In any case, this mono-objective analysis confirms that both objectives counter each other, thus a balance in the operating parameters must be achieved. As an expected conclusion of this analysis, the increase in temperature or processing time in thermal treatments favours the inactivation of the microorganism and disfavours (i.e., increases) acrylamide formation. However, the sensitivity of both responses to changes in temperature or time is significantly different.

### 3.2. Dynamics of Geobacillus Inactivation and Acrylamide Formation

**Figure 3:**
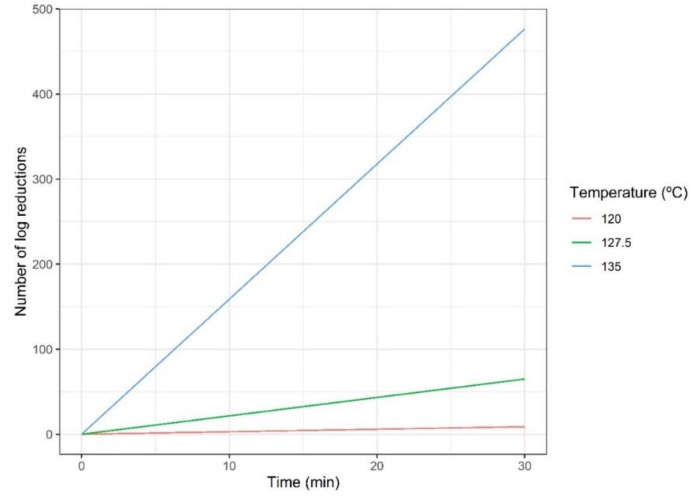
*Geobacillus stearothermophilus* inactivation dynamics according to the Bigelow model.

**Figure 4:**
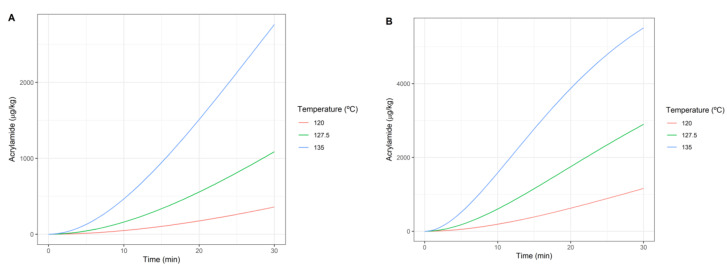
Acrylamide formation dynamics according to the Maillard reaction: potato puree (**A**) and prune juice (**B**).

As previously discussed, both objectives conflict. Therefore, to analyse the influence of the temperature on both objectives simultaneously, the inactivation rates and the amount of formed acrylamide for different treatment temperatures are evaluated. Simulated inactivation curves for the ranges of temperature and time considered are represented in Figure 3 following the Bigelow model (i.e., Equations (1) and (2)). The increase in temperature produces an increase in the slope of the inactivation curve and therefore a faster inactivation. On the other hand, acrylamide formation is explained by the Maillard reaction (Equation (3)) and its dynamics are represented in Figure 4. Figure 4A shows the formation rates for pureed potato. It is observed that the higher the temperature, the higher the formation. This is confirmed by Figure 4B, which corresponds to the amount of acrylamide formed in prunes juice.

**Figure 5:**
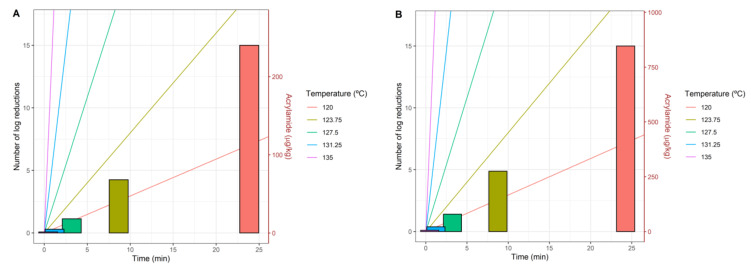
Inactivation rates represented as lines and acrylamide formed represented as bars for different temperatures when $P\left(log_{10}S\geq 6\right)=0.95$, potato puree (**A**) and prune juice (**B**).

Figure 5A,B represent the inactivation curves for five different temperatures between 120 and 135 °C and the acrylamide formed for each temperature when $P\left(log_{10}S\geq 6\right)=0.95$ for pureed potato and prune juice, respectively. Inactivation curves are represented as coloured lines and the left y-axis measures the number of logarithmic reductions. As discussed before, higher temperatures result in a higher slope of the inactivation rate. Bars placed at the times required for each treatment temperature show that processes at higher temperatures need less treatment time to achieve at least six log-reductions with a 95% probability. On the other hand, the height of each bar represents the acrylamide formed for each treatment (time–temperature). 

It is observed that both for the potato puree (Figure 5A) and the prune juice (Figure 5B), the lower the temperature, the greater the amount of acrylamide is formed due to the higher treatment time needed to achieve $\left(log_{10}S\geq 6\right)=0.95$. As the temperature increases, the treatment time required to inactivate the spores decreases, and therefore the amount of acrylamide formed is also lower. We must recall that both objectives (i.e., microbial inactivation and acrylamide formation) are determined by the combination of temperature and time. The processing time for each temperature is determined by the defined level of inactivation (i.e., $\left(log_{10}S\geq 6\right)=0.95$). This time is different for each temperature and the balance between dynamics explains the unexpected differences in the calculated acrylamide amount.

Table 2 quantifies the time and acrylamide formed for each temperature and three values of $P\left(log_{10}S\geq 6\right)$. For a probability of inactivation of 95%, if the temperature is increased by 12.5% (from 120 to 135 °C), the treatment time is reduced by 98% (from 23.92 to 0.46 min). Higher acrylamide amounts were observed for prune juice, as the amount of reducing sugars is higher than in pureed potato, although the qualitative appearance of the dynamics of the objectives is the same for both foods. The previously mentioned 12.5% increase in temperature (that would imply a 98% reduction in the treatment time) would result in a 99.5% and 99.3% reduction in acrylamide for pureed potato and prune juice, respectively. Therefore, as kinetics behave differently for temperature changes, an additional analysis to find the optimal trade-off between both objectives was performed, based on a multi-objective approach.

### 3.3. Multi-Objective Approach

The dependence of both objectives on time and temperature calls for a multi-objective optimization approach where the aim would be to find the pairs (temperature, time) that provide the Pareto front of optimal solutions (e.g., set of temperature/time solutions for which no objective can be improved without sacrificing the other one). However, the application of such an approach led to a set of Pareto solutions which consisted of the maximum temperature tested (135 °C) and different processing times (data not shown). This behaviour can be explained by the different dynamics of the objectives analysed above: an increase in temperature drastically reduces the processing time needed to achieve $P\left(log_{10}S\geq 6\right)=0.95$, which at the same time reduces the amount of acrylamide formed due to the slower dynamics of that process. For that reason, the non-dominated solutions consist of pairs of the maximum temperature tested and different processing times.

**Figure 6:**
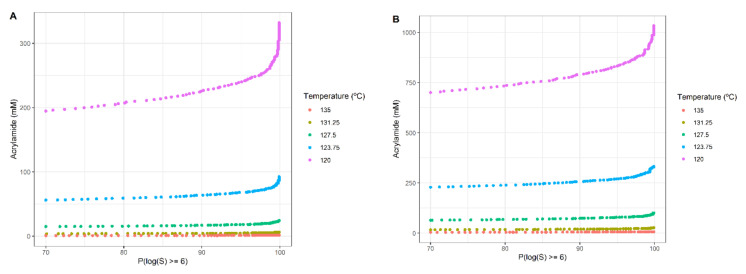
Acrylamide formed in a heat treatment that inactivates the microorganism for different values of $P\left(log_{10}S\geq 6\right)$: potato puree (**A**) and prune juice (**B**).

While this is a perfectly valid mathematical result, it is not so useful from the engineering point of view if we want to check the effects of other temperatures over the objectives, which can be critical in the case of considering additional objectives. For this reason, we eliminated the temperature as a decision variable and fixed it to some discrete values within the tested range, analysing the evolution of both objectives for each of the discrete temperatures over time. Figure 6 shows the acrylamide formed (y-axis) in a heat treatment that inactivates the microorganism for different values of $P\left(log_{10}S\geq 6\right)$, represented in the x-axis. 

The highest temperature tested (135 °C, orange dots) caused the lowest amount of formed acrylamide at any level of probability as treatment time was short. On the other hand, for the lowest temperature (associated with long treatment times, 120 °C, purple dots) the highest amount of acrylamide is formed. This reaffirms the results obtained in the previous section and also allows us to broaden the perspective of the problem, as it can be observed that increasing the probability of reaching the target microbial inactivation promotes the acrylamide formation, although with a very low sensitivity, except in the area of probability close to 100%, where the acrylamide formation tends to rise more significantly. The differences between foods (Figure 6A,B) are only manifested in the acrylamide levels, but the qualitative behaviour is similar.

For the lowest temperature (120 °C) the duration of the treatment exceeds 23 min for $P\left(log_{10}S\geq 6\right)=90\%$ (Table 2). On the other hand, increasing the temperature from 120 °C to 135 °C (+12.5%), the time needed for a 90% probability is around half a minute and acrylamide could be decreased by up to 99.5% and 99.3% for pureed potato and prune juice, respectively.

To increase $P\left(log_{10}S\geq 6\right)$ from 90% to 99%, the processing time must increase between approximately 10 and 13% depending on the temperature considered. Due to this variation, the amount of acrylamide increases by about 15–23% depending on the chosen temperature, considering both products again.

**Figure 7:**
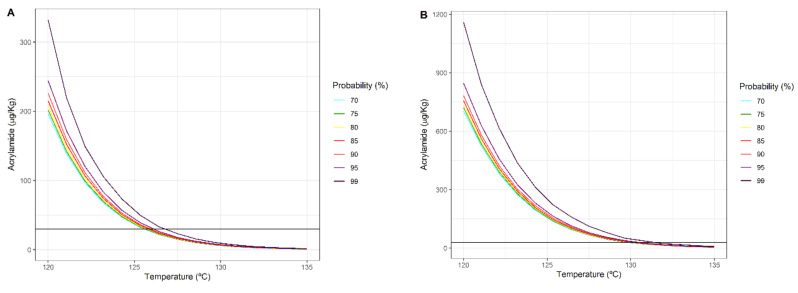
Acrylamide formed as a function of time for each temperature and $P\left(log_{10}S\geq 6\right)$: potato puree (**A**) and prune juice (**B**).

To assist the decision-making process, Figure 7A,B collect the necessary information to design a heat treatment that seeks to maximize food safety by inactivating a microorganism and minimizing acrylamide formation. These figures show the amount of acrylamide formed (y-axis) as a function of the temperature of the heat treatment (x-axis), as well as $P\left(log_{10}S\geq 6\right)$ (colour of the lines). The treatment time is determined by both the chosen temperature and the probability level, as explained below. The horizontal black line represents the maximum amount recommended in baby food 30 (μg/kg) [20], that was considered as a worst-case scenario. Figure 7A refers to pureed potato, whereas Figure 7B refers to prune juice. These figures provide a global vision of how the heat treatment directly determines the amount of formed acrylamide. The area that is above the horizontal line is an undesirable area, as the amount of acrylamide exceeds the recommendation. Ideally, we should remain in the lower region to ensure that the product has a low level of acrylamide while ensuring the inactivation of the microorganism given a defined probability value for $P\left(log_{10}S\geq 6\right)$. In both cases, it is observed that, when the temperature increases, acrylamide falls for this temperature range, as discussed above.

In Figure 7A, for pureed potato, the horizontal line divides the desirable region at around 126–127 °C for all the considered probabilities. Therefore, the heat treatments should be above that temperature. At 126–127 °C we would lie within the recommended limit of acrylamide (25–30 μg/kg) whereas an increase of 8–9 °C would produce around 1 μg/kg (Table 2). For the case of prune juice, Figure 7B, the same behaviour is observed. However, the amount of acrylamide formed is higher, as the initial concentrations of fructose and asparagine are also higher. In this case, the recommended limit for all the considered probabilities would lie within 129–130 °C. This information can be useful to consider the use of these foods in, e.g., baby-food products, to avoid exceeding the recommendation. 

Decision makers can use plots such as Figure 7 to decide which levels of acrylamide are likely to be present in the final product depending on$\;P\left(log_{10}S\geq 6\right)$ and temperature, which could affect other properties of the food not considered here. Another approach would be to select the desired acrylamide level and $P\left(log_{10}S\geq 6\right)$. In this way, the treatment temperature is determined. Figure 7 shows that, for high temperatures, as the duration of the treatments are very short regardless of the inactivation probability chosen, the acrylamide formation is low in every case. However, for lower temperatures the sensitivity is higher: small changes in temperature (linked to higher exposure times) result in significant changes in acrylamide production.

**Figure 8:**
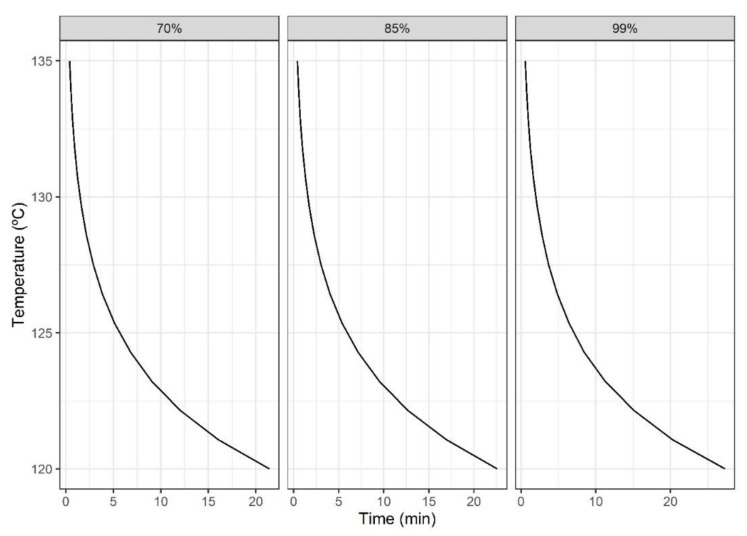
Treatment time required for each temperature and probability.

To complement these design steps, the duration of treatment should be calculated. For that purpose, Figure 8 shows the time (x-axis) for each temperature (y-axis) for three selected probability values of $P\left(log_{10}S\geq 6\right)$. Therefore, in the example, if the required temperature is 130 °C for a level of 85%, in the middle plot a treatment duration of 2.5 min is obtained. This value can be more easily retrieved by simulating both models (acrylamide production and microbial inactivation).

## 4. Conclusions

We have analysed the balance between microbial inactivation and acrylamide formation in the case where a thermoresistant microorganism can be present in food in which acrylamide can be formed due to high temperature thermal treatments. As a case study, we have chosen the inactivation of *Geobacillus stearothermophilus* in two particular foods (i.e., pureed potato and prune juice) that we have characterized with the Bigelow model. The acrylamide formation has been modelled with the Maillard equation. The analysis of the dynamics of both processes reveals that, to ensure a certain level of microbial inactivation, heat treatments at higher temperatures lead to decreased acrylamide formation, similar to the behaviour of quality components. This is due to the processes’ different sensitivities to temperature. While microbial inactivation is very sensitive (i.e., the times to produce a level of inactivation with a certain probability dramatically decreases with temperature), acrylamide formation is not. The methodology presented here can be used by decision makers to design heat treatments when food safety objectives are faced, maximizing inactivation and minimizing the amount of acrylamide (or other target substances). As the selected foods can be used as ingredients in baby foods, the obtained outcomes were compared with the EFSA’s recommendations about maximum acrylamide concentrations in baby food. Even at the highest temperatures, where the least amount of acrylamide is formed due to the short processing times to ensure the microbial inactivation, the expected acrylamide amounts are very close to the maximum EFSA’s recommendation, therefore it should be taken into account when using them in infant formulations.

The methodology presented here can be a basis to re-design processes where food safety and acrylamide formation are important issues. It can be used to make decisions when there are unexpected process variables deviations (e.g., lower treatment temperatures than expected). It can also be extended to other processes and microorganisms, and in future work we plan to include other conflicting objectives depending on temperature and time, such as quality or cost. This future work will also be addressed to experimentally validate the conclusions obtained here to refine the model fitting to other conditions and to check the effects of different food matrixes.

## Figures and Tables

**Table 1:** Initial concentrations of glucose, fructose, and asparagine in pureed potato and prune juice.

Concentration (mM)	Pureed Potato	Prune Juice
Glucose	10.740	213.426
Fructose	7.190	8.731
Asparagine	36.551	213.421

**Table 2:** Acrylamide formed and duration for different heat treatments and inactivation probabilities.

Temperature (°C)		90%			95%			99%	
	$Time\;\left(min\right)$	Acrylamide (μg/kg)		$Time\;\left(min\right)$	Acrylamide (μg/kg)		$Time\;\left(min\right)$	Acrylamide (μg/kg)	
		Pureed Potato	Prune Juice		Pureed Potato	Prune Juice		Pureed Potato	Prune Juice
120.00	23.15	226.52	789.23	23.92	241.29	829.20	25.66	275.45	913.46
123.75	8.43	64.94	257.49	8.71	68.14	269.85	9.28	77.56	300.61
127.50	3.11	17.43	73.78	3.22	18.15	78.79	3.47	21.06	87.71
131.25	1.16	4.50	19.70	1.21	4.74	21.38	1.31	5.55	23.79
135.00	0.44	1.14	5.18	0.46	1.21	5.61	0.49	1.40	6.23

## Supplementary Materials

The following are available online at https://www.mdpi.com/article/10.3390/foods10112535/s1, Figure S1: Experimental data and fitted model for the considered $log_{10}D/T$ values, Figure S2: Kinetic model for acrylamide formation from glucose, fructose and asparagine proposed by Knol et al. 2005. Supp_file1: Calculation of initial concentrations of glucose, fructose and asparagine for pureed potato and prune juice.
