# Supplementary material for: Dynamics of Microbial Inactivation and Acrylamide Production in High-Temperature Heat Treatments
Source: Foods. 2021 Oct 21;10(11):2535. doi: 10.3390/foods10112535 (PMC8624859; doi:10.3390/foods10112535)
Supplement: Supplementary file 1 [file foods-10-02535-s001.zip › foods-1396277-Supplementary.pdf]

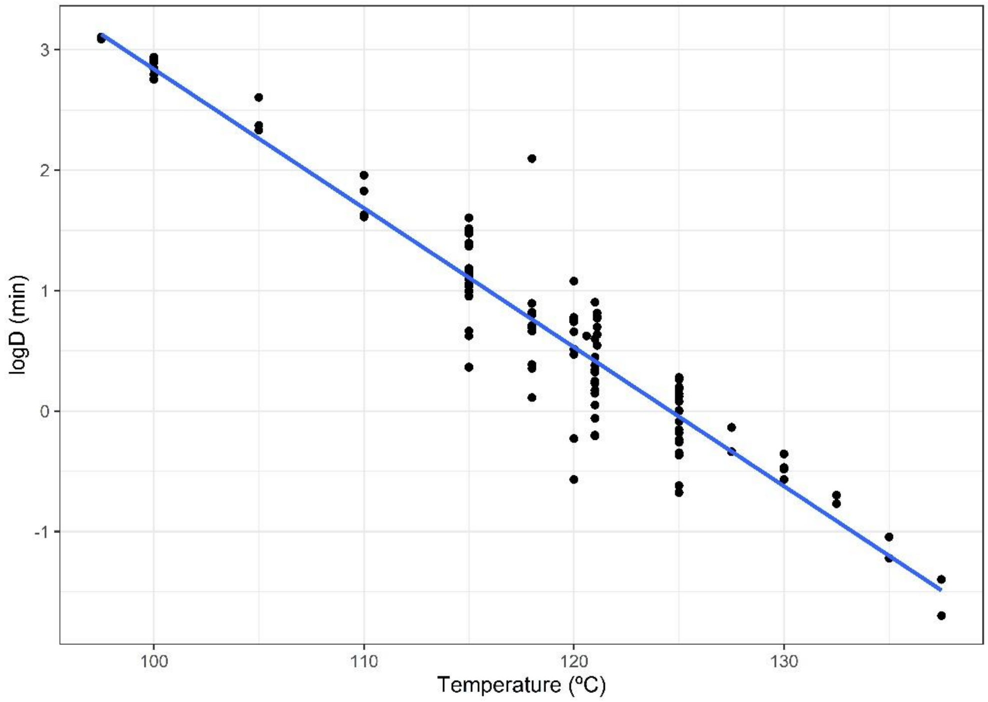

Figure S1. Experimental data and fitted model for the considered $log_{10}D/T$ values.

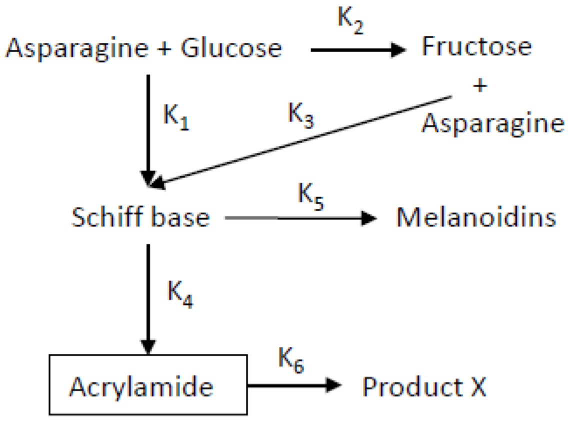

Figure S2. Kinetic model for acrylamide formation from glucose, fructose and asparagine proposed by Knol et al. 2005.

References

Knol, Jeroen J., Wil A.M. Van Loon, Jozef P.H. Linssen, Anne Laure Ruck, Martinus A.J.S. Van Boekel, and Alphons G.J. Voragen. 2005. "Toward a Kinetic Model for Acrylamide Formation in a Glucose-Asparagine Reaction System." *Journal of Agricultural and Food Chemistry* 53 (15): 6133–39. https://doi.org/10.1021/jf050504m.

Dynamics of microbial inactivation and acrylamide production in high temperature heat treatments.

Supplementary Material file 1: Calculation of initial concentrations of glucose, fructose and asparagine for pureed potato and prune juice.

The *red potato* variety is selected for the composition of the potato puree containing a significant quantity of glucose, asparagine and fructose of $16.94\, mmol/kg$, $57.65\, mmol/kg$ and $11.34\, mmol/kg$, respectively and because it is the variety that produces the highest amount of acrylamide (Vivanti et al., 2006). If the density of the potato puree is considered to be $634.01\, g/l$ (Ilhamto et al., 2014) then initial concentration of glucose is $C_{0,glucose} = 16.94 \frac{mmol}{kg} \cdot 0.63401 \frac{kg}{l} = 10.740\, mM$, initial concentration of asparagine is $C_{0,asparagine} = 57.65 \frac{mmol}{kg} \cdot 0.63401 \frac{kg}{l} = 36.551\, mM$ and initial concentration of fructose is $C_{0,fructose} = 11.64 \frac{mmol}{kg} \cdot 0.63401 \frac{kg}{l} = 7.190\, mM$ and .

On the other hand, in the case of prune juice, the amount of glucose, asparagine and fructose are, respectively $50\, mg/g$, $1.5\, mg/g$ and $50\, mg/g$, with a density of $0.769\, g/ml$ (Becalski et al., 2011). Since the molecular weights of glucose, asparagine and fructose are $180.156\, g/mol$, $132.12\, g/mol$ and $180.16\, g/mol$ respectively, initial concentration of glucose is $C_{0,glucose} = 50 \frac{mg}{g} \cdot 0.769 \frac{g}{ml} \cdot \frac{1}{180.156} \frac{mmol}{mg} \cdot 10^3 \frac{ml}{l} = 213.426\, mM$, initial concentration of asparagine is $C_{0,asparagine} = 1.5 \frac{mg}{g} \cdot 0.769 \frac{g}{ml} \cdot \frac{1}{132.12} \frac{mmol}{mg} \cdot 10^3 \frac{ml}{l} = 8.731\, mM$ and initial concentration of fructose is $C_{0,fructose} = 50 \frac{mg}{g} \cdot 0.769 \frac{g}{ml} \cdot \frac{1}{180.16} \frac{mmol}{mg} \cdot 10^3 \frac{ml}{l} = 213.421\, mM$.

References

Becalski, A., Brady, B., Feng, S., Gauthier, B. R., & Zhao, T. (2011). Formation of acrylamide at temperatures lower than 100°C: The case of prunes and a model study. *Food Additives and Contaminants - Part A Chemistry, Analysis, Control, Exposure and Risk Assessment*, *28*(6), 726–730. https://doi.org/10.1080/19440049.2010.535217

Ilhamto, N., Keller, H. H., & Duizer, L. M. (2014). The Effect of Varying Ingredient Composition on the Sensory and Nutritional Properties of a Pureed Meat and Vegetable. *Journal of Nutrition in Gerontology and Geriatrics*, *33*(3), 229–248. https://doi.org/10.1080/21551197.2014.927307

Vivanti, V., Finotti, E., & Friedman, M. (2006). Level of Acrylamide Precursors Asparagine, Fructose, Glucose, and Sucrose in Potatoes Sold at Retail in Italy and in the United States. *Journal of Food Science*, *71*(2), C81–C85. https://doi.org/10.1111/j.1365-2621.2006.tb08886.x
